# CardioMetAge: A Practical Clinical Aging Clock for Predicting Cardiometabolic Mortality and Morbidity

**DOI:** 10.1093/geroni/igaf122.2890

**Published:** 2025-12-31

**Authors:** Yucan Li, Xinming Xu, Kelin Xu, Xingdong Chen

**Affiliations:** Fudan University, Shanghai, Shanghai, China (People’s Republic); Fudan University, Shanghai, Shanghai, China (People’s Republic); Fudan University, Shanghai, Shanghai, China (People’s Republic); Fudan University, Shanghai, Shanghai, China (People’s Republic)

## Abstract

**Background:**

Existing biological age (BA) models primarily focus on systemic changes, overlooking alterations crucial for cardiometabolic diseases (CMDs).

**Methods:**

In this study, we developed the CardioMetAge model, a novel aging clock tailored to predict CMD-related outcomes. Trained in the NHANES-III, the model was applied to the continuous NHANES and UK Biobank. We evaluated it on the prediction of cardiometabolic mortality, morbidity, and disease state transitions. Its associations with proteomic pathways, lifestyle factors, and socioeconomic status, as well as the impact of caloric restriction intervention on its change, were also assessed.

**Results:**

The final form of CardioMetAge was concise, incorporating chronological age (CA) and 16 common clinical biomarkers. Its age deviation (CardioMetAgeDev) demonstrated stronger associations with CMD-related mortality, morbidity, and multi-morbidity than age deviations of traditional BA models. It also outperformed CA and PhenoAge in predicting 10-year incidence risks for CMDs. Beyond prediction, our findings highlighted the biological determinants of cardiometabolic aging, with proteomic analyses linking CardioMetAgeDev to inflammatory activation and metabolic disorders. Analysis of modifiable factors revealed that lifestyle and socioeconomic status influenced CMD risks, partly through their effect on CardioMetAgeDev, with mediation proportions of 30.3% and 11.1%, respectively. Additionally, two-year caloric restriction slowed the progression of CardioMetAge by 0.92 years (95% CI: 0.27 to 1.57) compared to the ad libitum group.

**Conclusion:**

CardioMetAge outperformed existing BA models in simplicity and in predicting CMD outcomes. It provides valuable insights into the mechanisms of cardiometabolic aging and holds potential for clinical monitoring and evaluating the effectiveness of interventions.

